# Automation of Lung Ultrasound Interpretation via Deep Learning for the Classification of Normal versus Abnormal Lung Parenchyma: A Multicenter Study

**DOI:** 10.3390/diagnostics11112049

**Published:** 2021-11-04

**Authors:** Robert Arntfield, Derek Wu, Jared Tschirhart, Blake VanBerlo, Alex Ford, Jordan Ho, Joseph McCauley, Benjamin Wu, Jason Deglint, Rushil Chaudhary, Chintan Dave, Bennett VanBerlo, John Basmaji, Scott Millington

**Affiliations:** 1 Division of Critical Care Medicine, Western University, London, ON N6A 5C1, Canada; Chintan.Dave@lhsc.on.ca (C.D.); john.basmaji@lhsc.on.ca (J.B.); 2 Schulich School of Medicine and Dentistry, Western University, London, ON N6A 5C1, Canada; dwu2021@meds.uwo.ca (D.W.); jtschirhart2024@meds.uwo.ca (J.T.); jho2021@meds.uwo.ca (J.H.); rchaud@uwo.ca (R.C.); 3 Faculty of Mathematics, University of Waterloo, Waterloo, ON N2L 3G1, Canada; bvanberlo2021@meds.uwo.ca; 4 Independent Researcher, London, ON N6A 1L8, Canada; aford3532@gmail.com; 5 Faculty of Engineering, University of Waterloo, Waterloo, ON N2L 3G1, Canada; joewmccauley@gmail.com; 6 Independent Researcher, London, ON N6C 4P9, Canada; benjamin.dq.wu@gmail.com; 7 Faculty of Systems Design Engineering, University of Waterloo, Waterloo, ON N2L 3G1, Canada; jason.deglint.engr@gmail.com; 8 Faculty of Engineering, University of Western Ontario, London, ON N6A 5C1, Canada; bennettjlvb@gmail.com; 9 Department of Critical Care Medicine, University of Ottawa, Ottawa, ON K1N 6N5, Canada; scottjmillington@gmail.com

**Keywords:** deep learning, ultrasound, lung ultrasound, artificial intelligence, automation, imaging

## Abstract

Lung ultrasound (LUS) is an accurate thoracic imaging technique distinguished by its handheld size, low-cost, and lack of radiation. User dependence and poor access to training have limited the impact and dissemination of LUS outside of acute care hospital environments. Automated interpretation of LUS using deep learning can overcome these barriers by increasing accuracy while allowing point-of-care use by non-experts. In this multicenter study, we seek to automate the clinically vital distinction between A line (normal parenchyma) and B line (abnormal parenchyma) on LUS by training a customized neural network using 272,891 labelled LUS images. After external validation on 23,393 frames, pragmatic clinical application at the clip level was performed on 1162 videos. The trained classifier demonstrated an area under the receiver operating curve (AUC) of 0.96 (±0.02) through 10-fold cross-validation on local frames and an AUC of 0.93 on the external validation dataset. Clip-level inference yielded sensitivities and specificities of 90% and 92% (local) and 83% and 82% (external), respectively, for detecting the B line pattern. This study demonstrates accurate deep-learning-enabled LUS interpretation between normal and abnormal lung parenchyma on ultrasound frames while rendering diagnostically important sensitivity and specificity at the video clip level.

## 1. Introduction

Lung ultrasound (LUS) is a versatile thoracic imaging method that offers the diagnostic accuracy of a CT scan for many common clinical findings, with all the advantages of portable, handheld technology [1,2,3,4]. Since recent reports have highlighted that the potential for LUS dissemination is near-limitless, for example, primary care, community settings, developing countries, and outer space [5,6,7], accordingly, it has been praised as a worthy upgrade to auscultation [8]. With experts in its use in persistent short supply [9,10,11,12], solutions for automating the interpretation of LUS form the most probable method to ensure maximal access to the unique offerings of this technique.

One of the most popular automation techniques for imaging is deep learning (DL), which has been applied to a wide variety of imaging including retinal fundal photographs, echocardiography, computed tomography (CT), and chest radiographs for identification of diabetic retinopathy, cardiac structure and function, COVID-19, and pulmonary nodules [13,14,15,16]. The diagnostic accuracy of DL for diagnosis of respiratory disease using chest radiograph or CT has been extensively studied [17] and proven to be highly effective. As in other imaging techniques, the use of DL to construct computer vision classifiers for automated interpretation can be relied on to accelerate access to the benefits of LUS.

However, DL work with LUS is immature due to small, poorly labelled datasets arising from the point-of-care, rather than diagnostic, workflow of LUS that is less amenable to archiving and formal reporting. The current LUS literature has been limited to COVID-19 applications [18,19,20] and small datasets [21,22]; the largest study, to date, utilized 400 unique clips further split into subclips to augment data, and lacked generalizability as data was sourced from a single institution. In addition, these studies did not capture the metadata of ultrasound clips, such as machine and probe characteristics, which can serve as information for feature and error analysis. These would provide insight to inform targeted data collection and model re-training to further improve the generalizability of a DL model.

With the goal of increasing accessibility, aiding widespread deployment, and enhancing the efficiency of LUS application in clinical settings, our work applies DL techniques to LUS interpretation, beginning with clinically identifiable features. The distinction between A lines (normal aeration [23]) and B lines (alveolar-interstitial syndrome [24]) on LUS is clinically important, forming the backbone of multiple clinical decision trees for real-time respiratory diagnoses and treatment choices [4,25,26,27,28,29]. As such, a DL solution for this task would be highly appropriate for an automated LUS interpretation strategy. Employing a large archive of well-labelled, local LUS images for DL training as well as an external dataset for validation, we sought to develop a robust, generalizable deep learning solution to address the binary distinction between A lines and B lines with LUS.

In the proposed two-part DL solution presented in this paper, first, we develop a frame-based, A line vs. B line classifier with multicenter LUS still frames. Following this, approximating the continuous nature of how clinicians perceive and interpret LUS, the diagnostic performance of this classifier against multicenter LUS clips is studied. With a focus on sensitivity and specificity, we characterize how these diagnostic parameters may be maximized as well as selectively prioritized to suit varying clinical environments where automated LUS might be considered.

## 2. Methods

### 2.1. Dataset Curation and Labelling

#### 2.1.1. Local Data

**Figure 1:**
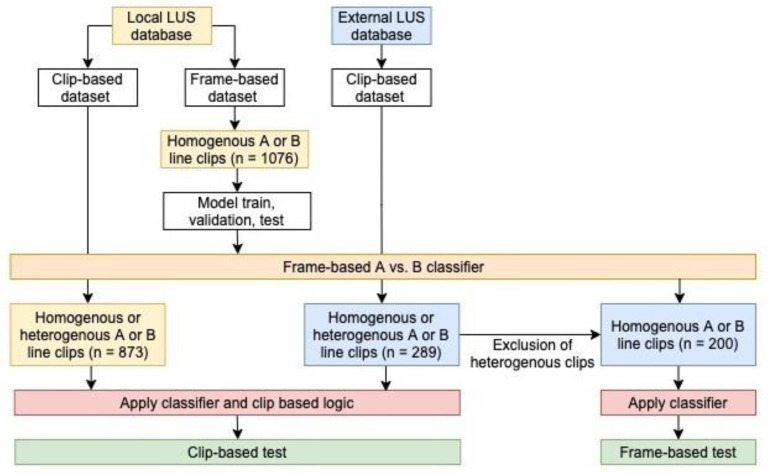
Flowchart of dataset creation, labelling, data allocation, and model training used in the study.

Using our institutional point-of-care ultrasound database (Qpath E, Port Coquitlam, BC, Canada), all LUS exams archived from all clinical environments since 2012 were downloaded to a local drive in mp4 format. Due to the large volume of data (over 120,000 LUS clips), our dataset for this project was a subset of LUS studies whose size was determined by the labelling workflow (see below) and the timeline of this project. Figure 1 outlines the study workflow.

#### 2.1.2. External Data

Exams labelled as LUS studies within the University of Ottawa ultrasound archiving system (Qpath, Port Coquitlam, BC, Canada) were exported from 50 patients to a shared drive.

#### 2.1.3. Data Labelling

**Figure 2:**
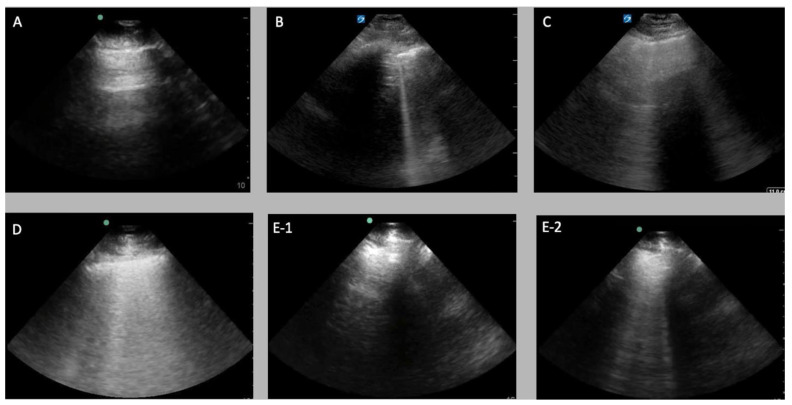
Still image representations of the various lung ultrasound artifacts, severity and heterogeneity applied as labels to our data: (**A**) A-line pattern (normal lung) class; (**B**) fewer than 3 B lines class; (**C**) moderate B line class; (**D**) severe B line class; (**E**) 2 frames showing heterogeneity within the same clip with A lines (**E-1**) followed by moderate B lines (**E-2**) sliding in to view.

LUS clips were uploaded to an online platform (Labelbox, San Francisco, CA, USA) for vetting and labelling. Clips that had text over the image, cardiac structures in view, or included abdominal organs and/or the diaphragm (pleural views) were excluded from this analysis. All other clips were labelled with either an A line or a B line identity. The B line label contained the following 3 sublabels: (1) mild, fewer than 3 B lines; (2) moderate, occupying less than 50% of the pleural line; and (3) severe, occupying >50% of the pleural line. The clip-level B line label was additionally stratified as either homogeneous (all frames of the clip contained B lines) or heterogeneous (B lines seen in some frames but not others, coming in and out of view with tidal respiration). This distinction would allow homogeneous clips to be the source of the frame-based data for our classifier training, because the overall clip label (“B lines”) was valid across all individual frames. Heterogeneous B line clips would be used in clip-level inference and validation, as outlined below. See Figure 2 and Videos S1–S5 in Supplementary Materials for examples of each label.

Local data labels were generated by clinical members of our team (labelling training methods in Table S1 in the Supplementary Materials) and reviewed by an expert in LUS (R.A.), while external data were subjected to dual expert (R.A. and S.M.), independent, blinded labelling. This latter approach was taken given the importance of external data serving as a validation set.

### 2.2. Experimental Setup

#### 2.2.1. Frame-Based Data

**Figure 3:**
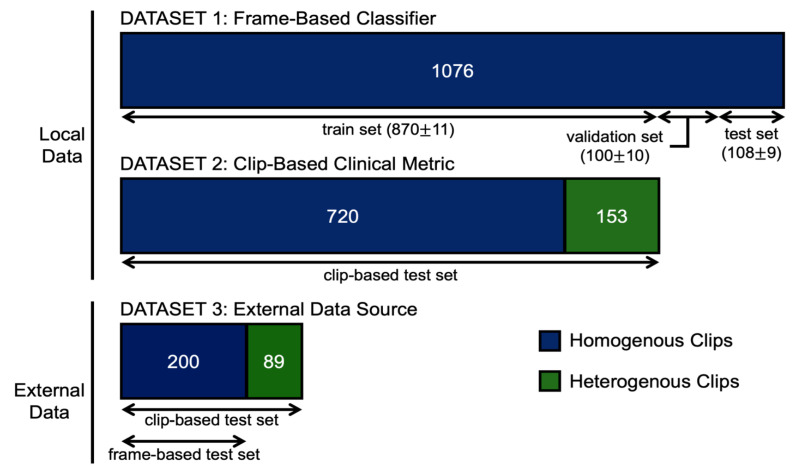
Schematic breakdown of the data sources, data volume, hetero/homogeneity, and how data were allocated in our frame-based classifier development and clip-based clinical metric. For the frame-based classifier, the number of clips used for the training, validation, and test sets are presented as the mean ± standard deviation of the ten-fold cross-validation partitions.

All local homogeneous A or B line clips labelled prior to 15 May 2021 were used for frame-based classifier training and validation. Locally, 723 homogenous A line clips and 353 homogenous B line clips met criteria. The external data yielded 92 homogenous A line clips and 108 homogenous B line clips. Table 1 provides further dataset details while Figure 3 offers a schematic representation of our data volumes and how they were used.

#### 2.2.2. Clip-Based Inference Data

The local clip inference data were generated from a combination of all heterogeneous A or B line data clips and all homogeneous clips generated from our labelling team after the frame-based classifier was already trained, thus, avoiding data leakage between the frame-based training data and clip-inference data (as this may inflate performance). Locally, there were 523 A line clips and 350 B line clips. Among the B line clips, 153 were heterogeneous. The external clip inference dataset was screened similarly yielding 92 A line clips and 197 B line clips. Among the B line clips, 89 were heterogeneous. Details regarding these datasets are in Table 2.

#### 2.2.3. Dataset Split

Prior to a training experiment, the dataset was randomly split into training, validation, and test sets by patient ID. Therefore, all the clips obtained from each unique patient were confined to a single set (i.e., training, validation, or test) without overlap. A summary of the split used in K-fold cross-validation are outlined in Table 3.

#### 2.2.4. Data Preprocessing

**Figure 4:**
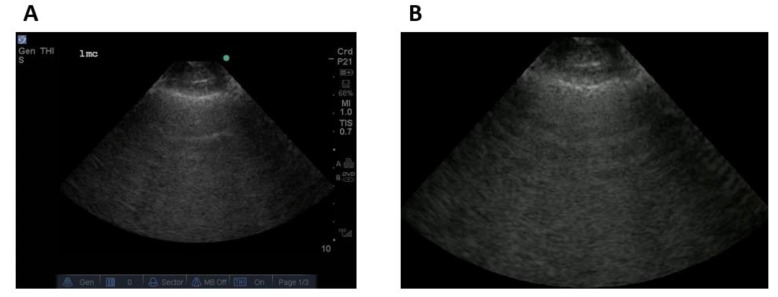
Masking of native ultrasound image (**A**) resulting in a frame consisting of only the ultrasound image without extraneous screen markings (**B**).

All ultrasound clips were deconstructed into their constituent frames. Following this, the frames were scrubbed of all on-screen information (e.g., vendor logos, battery indicators, index mark, and depth markers) extraneous to the ultrasound beam itself (see Figure 4). This was done using a dedicated deep learning masking software for ultrasound (AutoMask, WaveBase Inc., Waterloo, ON, Canada).

Transformations were stochastically applied to training batches as a means of data augmentation. Possible transformations included rotation up to 45° clockwise or counterclockwise, vertical or horizontal width shifting up to 10%, magnification up to 10% inwards or outwards, shear up to 10° counterclockwise, horizontal reflection and brightness increase/decrease up to 30%. These methods were applied to increase the heterogeneity of the training dataset because, despite a large quantity of frames, the number of distinct clips and individual patients was comparatively lower.

### 2.3. Frame-Based Deep Learning Classifier

#### Model Architecture

**Figure 5:**
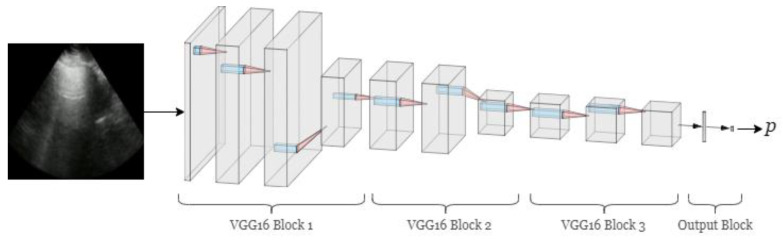
Neural network model architecture. The model consists of the first 3 blocks of VGG16. Each VGG16 block is a series of single-stride convolutions with 3 × 3 filters, followed by a 2 × 2 maxpool operation. The maxpool layer of the third block is removed from our model. The output block consists of a global average pooling layer, followed by dropout and a 2-node fully connected layer. The softmax activation function is applied to the final layer, producing the final prediction probabilities.

After iterative experiments with a subset of our data on feedforward convolutional neural networks (CNNs), residual CNNs, and benchmark CNN architectures pretrained on ImageNet [30], we chose a model comprised of the first 3 blocks of VGG16 as our network architecture (Figure 5, Table S2 in the Supplementary Materials) [31]. This architecture exploits the pretrained, earlier layers of VGG16 for low-level features (e.g., edges and lines), while avoiding more sophisticated feature detection is likely unhelpful to interpreting lower complexity LUS images. Additionally, this approach afforded a lighter computational demand and may be less prone to overfitting the training data than the full VGG16 architecture.

The model’s prediction is a probability distribution indicating its confidence that an input lung ultrasound frame exhibits A lines or B lines. We elected to focus on frame-based predictions, as single LUS frames are able to convey A vs. B line patterns and represent the building block unit of clips. Therefore, a classifier at the frame level has the greatest agility to be applied to clips of varying compositions as is typical of point-of-care imaging.

The prediction for a single frame is the probability distribution $p=\left[p_{A},\;p_{B}\right]$ obtained from the output of the softmax final layer, and the predicted class is the one with the greatest probability (i.e., argmax(p)) (full details of the classifier training and evaluation are provided in the Methods section, Table S3 of the Supplementary Materials).

### 2.4. Clip-Based Clinical Metric

As LUS is not experienced and interpreted by clinicians in a static, frame-based fashion, but rather in a dynamic (series of frames/video clip) fashion, mapping the classifier performance against clips offers the most realistic appraisal of eventual clinical utility. Regarding this inference as a kind of diagnostic test, sensitivity and specificity formed the basis of our performance evaluation [32].

We considered and applied multiple approaches to evaluate and maximize performance of a frame-based classifier at the clip level. For clips where the ground truth is homogeneously represented across all frames (e.g., a series of all A line frames or a series of all B line frames), a clip averaging method would be most appropriate. However, with many LUS clips having heterogeneous findings (where the pathological B lines come in and out of view and the majority of the frames show A lines), clip averaging would lead to a falsely negative prediction of a normal/A line lung (see the Supplementary Materials for the methods and results—Figures S1–S4 and Table S6 of clip averaging on our dataset).

To address this heterogeneity problem, we devised a novel clip classification algorithm which received the model’s frame-based predictions as input. Under this classification strategy, a clip is considered to contain B lines if there is at least one instance of 𝜏 contiguous frames for which the model predicted B lines. The two hyperparameters defining this approach are defined as follows:

**Classification threshold (*t*)** The minimum prediction probability for B lines required to identify the frame’s predicted class as B lines.

**Contiguity threshold (*𝜏*)** The minimum number of consecutive frames for which the predicted class is B lines.

Equation (1) formally expresses how the clip’s predicted class $\hat{y}\in \left\{0,\;1\right\}$ is obtained under this strategy, given the set of frame-wise prediction probabilities for the B line class,$\;P_{B}=\left\{p_{B_{1}},\;p_{B_{2}},\;\ldots ,\;p_{B_{n}}\right\}$, for an *n*-frame clip. Further details regarding the advantages of this algorithm are in the Methods section of the Supplementary Materials.

Equation (1):

(1) $$\hat{y}={𝟙}_{\vee_{i=1}^{n-\tau +1}\left[\wedge_{j=i}^{j+\tau -1}\left[p_{B_{j}}\geq t\right]\right]}\left(P_{B}\right)$$

We carried out a series of validation experiments on unseen internal and external datasets, varying both of these thresholds. The resultant metrics guided the subsequent exploration of the clinical utility of this algorithm.

### 2.5. Explainability

We applied the Grad-CAM method [33] to visualize which components of the input image were most contributory to the model’s predictions. The results are conveyed by color on a heatmap, overlaid on the original input images. Blue and red regions correspond to the highest and lowest prediction importance, respectively.

## 3. Results

### 3.1. Frame-Based Performance and K-Fold Cross-Validation

**Figure 6:**
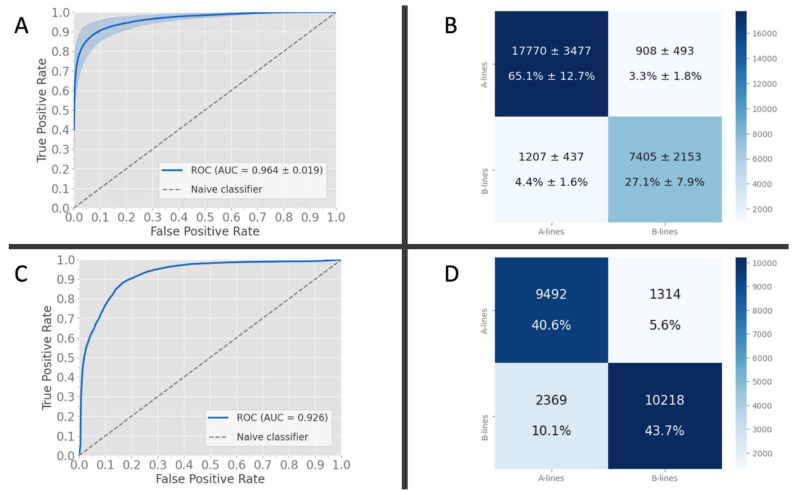
Receiver-operator characteristic curves and confusion matrices for the local (**A**,**B**) and external (**C**,**D**) data. (**A**) AUC of the k-fold validation internally gave an average of 0.96 (±0.02) with the corresponding confusion matrix results in (**B**); (**C**) AUC of the frame-based inference on the external data with our trained classifier yielded an AUC of 0.926 with the corresponding confusion matrix in (**D**).

Our K-fold cross-validation yielded a mean area under (AUC) the receiver operating curve of 0.964 for the frame-based classifier on our local data (Figure 6, Panel A). The confusion matrix of frame-wise predictions exhibits a strong diagonal pattern (Figure 6, Panel B). A summary of the results is shown in Table 4 (full results in Table S5 in the Supplementary Materials).

### 3.2. Frame-Based Performance on External Data

The AUC obtained from the external data at the frame level was 0.926 (Figure 6, Panel C). The confusion matrix (Figure 6, Panel D) of frame-wise predictions exhibit a strong diagonal pattern, supporting the results of the individual class performance.

A summary of the results is shown in Table 4.

### 3.3. Explainability

**Figure 7:**
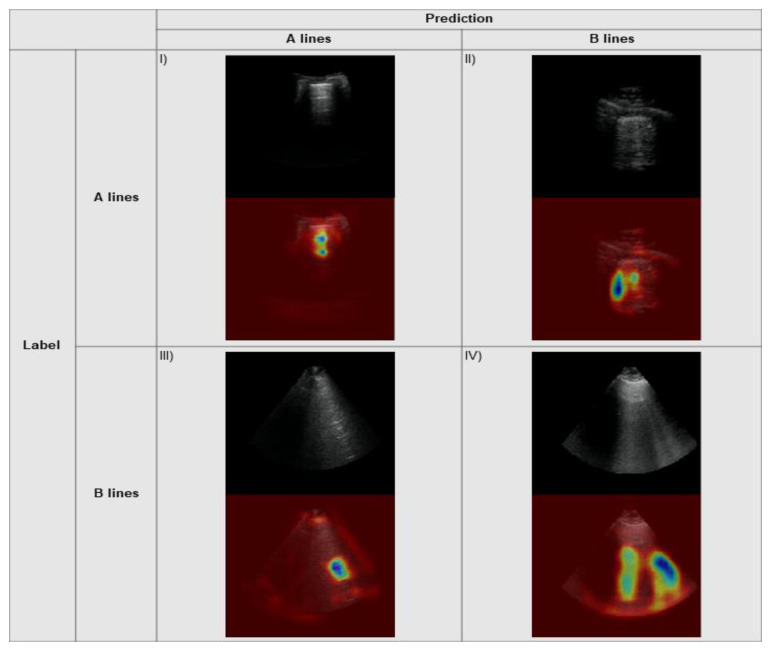
Grad-CAM heatmaps overlying example frames from our local data: (**I**) Correctly predicted A line frame with a prediction probability of 0.96; (**II**) A line frame incorrectly predicted as a B line frame with a prediction probability of 0.69 for B line; (**III**) B line frame incorrectly predicted as an A line frame with a prediction probability of 0.86 for A line; (**IV**) Correctly predicted B line frame with a prediction probability of 1.00.

**Figure 8:**
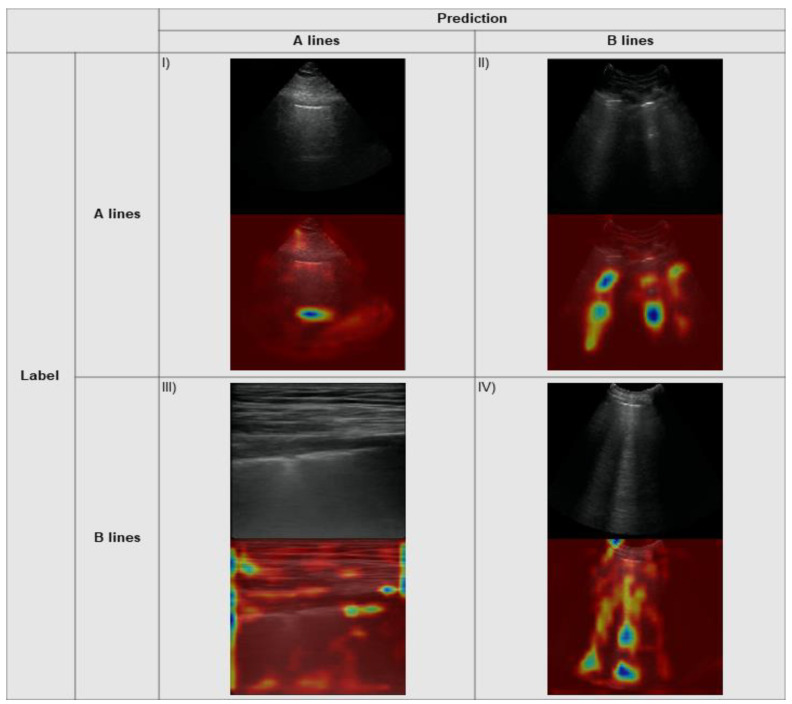
Grad-CAM heatmaps overlying example frames from the Ottawa data: (**I**) Correctly predicted A line frame with a prediction probability of 0.95; (**II**) A line frame incorrectly predicted as a B line frame with a prediction probability of 0.99 for B line; (**III**) B line frame incorrectly predicted as an A line frame with a prediction probability of 0.75 for A line; (**IV**) Correctly predicted B line frame with a prediction probability of 1.00.

The Grad-CAM explainability algorithm was applied to the output from the model on our local test set data and the external data. Example heatmaps with associated predictions are seen for our internal data and external data in Figure 7 and Figure 8, respectively. The correctly predicted A line frames demonstrate strong activations on the horizontal markings, indicating the correct areas where a clinician would assess for this specific pattern. Similarly, there are strong activations along the vertically oriented B lines on the correctly identified clips for this pattern. The incorrectly predicted frames show activations taking on a similar morphology for the predicted class (i.e., horizontal shapes for predicted A lines, vertical shapes for predicted B lines).

### 3.4. Clip-Based Clinical Metric

**Figure 9:**
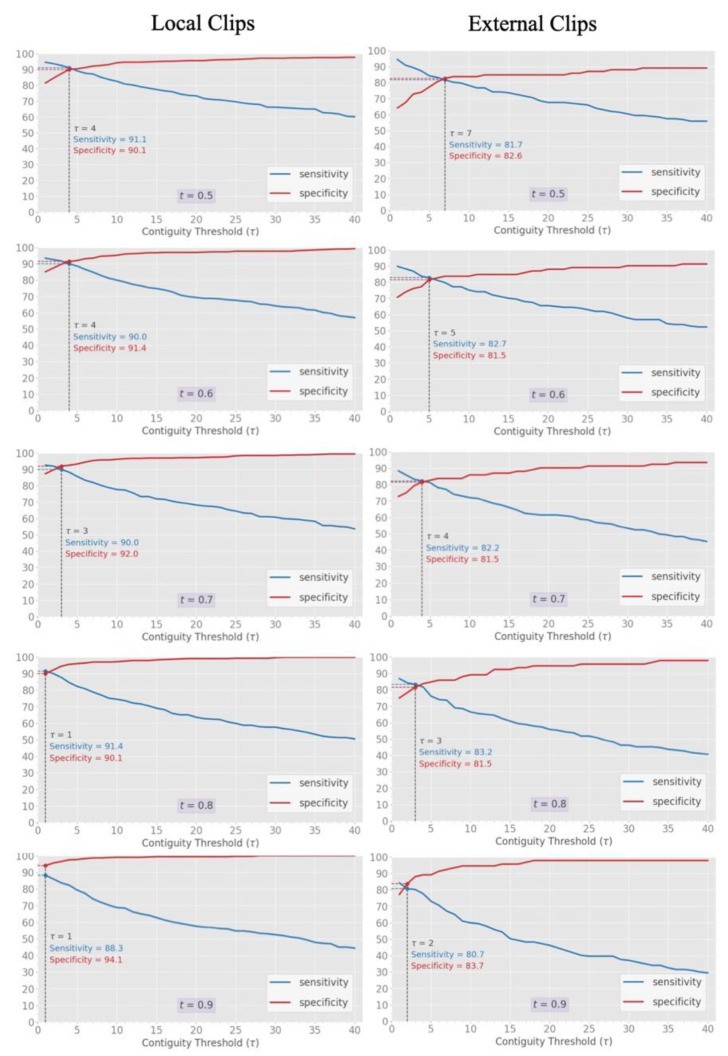
Influence of varying the contiguity threshold (T) and the classification threshold (t) across the internal and external clip-based data. While absolute diagnostic performance (intersection of sensitivity and specificity, dashed line) differed between the internal and external set, common trends in increasing both T and t were seen. Increases in T at lower levels of t are useful to increase performance at all levels for external data while this effect plateaus at a t of 0.8 for local data.

The relationship of a contiguity threshold (T) from 1 to 40 and/or a frame classification threshold (t) from 0.5 to 0.9 to diagnostic sensitivity and specificity was fully explored on both internal and external clip-based inference datasets (Figure 9). In both datasets, increasing t led to incremental upward translation of the specificity curve with modest negative translation of the sensitivity curve. With a T > 1 for any given t, specificity was able to be further augmented with modest reductions in sensitivity. Across all thresholds, peak diagnostic performance, as determined by optimum combined sensitivity and specificity, was a t of 0.7 and a T of 3 (sensitivity of 90.0% and specificity of 92.0%) for the internal data, and a t of 0.8 and a T of 3 (sensitivity of 83.2% and specificity of 81.5%) for the external data.

## 4. Discussion

In this study, we developed a deep learning solution for accurate distinction between the A line and B line pattern on lung ultrasound. Since this classification, between normal and abnormal parenchymal patterns, is among the most impactful and well-studied applications of LUS, our results form an important step toward the automation of LUS interpretation.

With reliable frame level classification (local AUC of 0.96, external AUC of 0.93) and explainability figures that show appropriate pixel activation regions, results support generalized learning of the A line and B line pattern. Clip-level application of this model was carried out to mimic the more difficult, clinical task of interpreting LUS in a real-time, continuous fashion at a given location on the chest.

A challenge of classifying B lines at the clip level is to ensure sufficient responsiveness that low burden B line clips (either because of flickering, heterogeneous frames, or a low number of B lines) are accurately identified, while still preserving specificity to the classifier. The thresholding techniques we devised around frame prediction strength and contiguity of such predictions were successful in addressing this challenge, while also providing insight into how an A vs. B line classifier may be customized for a variety of clinical environments. Through adjustment of these thresholds (Figure 9), varying clinical use cases may be matched with appropriate emphasis on either greater sensitivity or specificity. Further considerations such as disease prevalence, presence of disease specific risk factors, and the results and/or availability of ancillary tests and expert oversight would also influence how automated interpretation should be deployed [34].

Among the many DL approaches to be considered for medical imaging, our frame-based foundation was chosen deliberately for the advantages it may offer for eventual real-time automation of LUS interpretation. Larger, three-dimensional or temporal DL models that might be applied to perform clip-level inference would be too bulky for eventual front-line deployment on the edge and also lack any semantic clinical knowledge that our clip-based inference approach is intended to mimic.

The automation of LUS delivery implied by this study may seem futuristic amid some public trepidation about deploying artificial intelligence (AI) in medicine [35]. Deep learning solutions for dermatology [36] and for ocular health [37], however, have shown tolerance exists for non-expert and/or patient-directed assessments of common medical concerns [38]. As acceptance for AI in medicine grows, automated LUS may be anticipated to satisfy the consistently exceptional demand for lung imaging [39], especially where access to standard imaging may not be convenient or possible. The recently announced reimbursement for DL-enhanced imaging in the United States will, by offsetting the costs of developing such solutions, accelerate interest in the DL-imaging interface [40]. Beyond A and B lines, LUS automation priorities can be expected to include lung sliding, pleural effusion, and consolidation. Additionally, multicenter validation of automated diagnosis [19] or prognosis [18] with LUS offers promising research avenues.

Real world deployment of a classifier as we have developed will require further progress before it can be realized. Firstly, since LUS is user dependent, a method of standardizing acquisition, as has recently been proposed, can only enhance the opportunities for both DL development and implementation in LUS [41]. Anticipating that technical standards take significant time to be adopted, however, a more realistic approach may be to pair automated interpretation with image guidance systems that assure standards that meet the needs of the image classifier. Such an approach has recently been described with some success in the domain of AI-assisted echocardiography [42]. The other barrier to deployment is how to run the DL technology “on the edge” at the patient’s bedside with a portable machine capable of LUS. Eventual integration of high-performance GPUs with ultrasound devices will address this; however, in the interim, portable “middleware” devices capable of interacting directly with ultrasound machines and running AI models in real time have been developed and are commercially available [43].

Despite the rarity of DL work with LUS, there have been some recent studies that have addressed LUS [20,21,22,44]. These studies, with a wide array of different DL approaches, all share a non-clinical emphasis and small datasets. Our work differs significantly through a comparatively much larger LUS data volume from multiple centers, rigorous curation and labelling methods that resemble reference standards [45], and a pragmatic, clinical emphasis on diagnostic performance. In addition, while medical DL classifiers have struggled notoriously with generalization [46,47], our model performed well on an external dataset with reasonably distinct acquisition features as compared with our data.

There are important limitations to our work. The implicit heterogeneity of point-of-care data can contribute to unseen learning points for our model that could unduly increase performance. We have sought to mitigate these effects through rigorous preprocessing as well as through our K-fold validation methods, external validation, and explainability. Despite generalizable results against the external data set, a performance gap at the frame and clip level was seen. False positive B line predictions (B line prediction for ground truth A line clips, Figure 9, and in Supplementary Materials, Figure S2) provided the greatest challenge to our model and was driven largely by dataset imbalances relative to the training data: images generated with either curved linear probe, cardiac preset, or the Philips machine. This understanding will inform future iterations of this classifier. While we have designed our classifier as a “normal vs. abnormal” model, there is an opportunity for greater granularity within the B line class. Features such as subpleural consolidations and pleural line irregularity [48] were not addressed by this classifier. Combining the present model with other published models devoted to disease-specific diagnoses within the B line class seems desirable [19].

## 5. Conclusions

The information presented here supports an eventual goal of automated LUS through deep learning. We describe the development of an accurate A vs. B line, frame-based classifier validated at the clip level. Novel techniques to both maximize and adjust diagnostic performance to suit the priorities of differing clinical environments have further been established. Future work will rely on broader data representation and evaluating the feasibility and accuracy of real-time clinical deployment.

## Figures and Tables

**Table 1:** Homogeneous ultrasound data characteristics used for frame-based classifier training and validation. ED, emergency department; ICU, intensive care unit.

	Local Data		External Data	
Clip Label	A lines (normal class)	B lines (abnormal class)	A lines (normal class)	B lines (abnormal class)
Patients	253	155	40	32
Frames	186,772	86,119	10,806	12,587
# of clips	723	353	92	108
Average clips per patient	2.86	2.28	2.3	3.375
Female sex/total	84/253	66/155	12/40	8/32
Unknown gender	19	7	6	8
Mean age (STD)	63.23 (17.17)	66.76 (16.11)	62.26 (16.72)	66.5 (11.51)
Machine Vendors	Sonosite: 721 Mindray: 2	Sonosite: 353	Philips: 62 SonoSite: 30	Philips: 37 SonoSite: 71
Transducers	Phased array: 596 Curved linear: 119 Linear: 8	Phased array: 319 Curved linear: 30 Linear: 4	Phased array: 46 Curved linear: 22 Linear: 24	Phased array: 66 Curved linear: 23 Linear: 19
Imaging Preset	Abdominal: 671 Lung: 33 Vascular: 4 Cardiac: 15	Abdominal: 309 Lung: 20 Cardiac: 14 Obstetrical: 7 Other: 3	Abdominal: 10 Lung: 35 Cardiac: 26 Nerve: 8 FAST: 7 Vascular: 6	Abdominal: 11 Lung: 20 Cardiac: 55 Nerve:1 FAST: 4 Superficial: 3 Vascular: 14
Location (by patient)	ICU: 166 ED: 82 Ward: 5	ICU: 124 ED: 26 Ward: 5	ICU: 21 ED: 14 Ward: 5	ICU: 19 ED: 6 Ward: 7
Depth (STD, cm)	11.56 (3.48)	12.50 (3.43)	11.28 (4.64)	11.13 (3.88)

**Table 2:** Data characteristics for clip-based inference ultrasound clips. ED, emergency department; ICU, intensive care unit.

	Local Data		External Data	
Clip Label	A lines (normal class)	B lines (abnormal class)	A lines (normal class)	B lines (abnormal class)
Patients	156	120	40	49
Clips	523	350	92	197
Average clips per patient	2.35	1.92	2.30	4.02
Heterogeneous	153/873		89/289	
Female sex/total	96/151	55/118	13/40	16/49
Unknown gender	5	2	5	8
Mean age (STD)	59.92 (17.19)	64.19 (16.84)	62.51 (16.54)	65.29 (13.65)
Machine Vendors	SonoSite: 516 Mindray: 4 Philips: 3	SonoSite: 349 Philips: 1	Philips: 62 Sonosite: 30	Philips: 90 Sonosite: 107
Transducers	Phased array: 448 Curved linear: 67 Linear: 8	Phased array: 308 Curved linear: 33 Linear: 9	Phased array: 46 Curved linear: 22 Linear: 24	Phased array:127 Curved linear:43 Linear: 27
Imaging preset	Abdominal: 463 Cardiac: 21 Lung: 33 MSK: 1 Vascular: 6	Abdominal: 312 Cardiac: 11 Lung: 25 Vascular: 2	Abdominal: 10 Cardiac: 26 FAST: 7 Lung: 35 Nerve: 8 Vascular: 6	Abdominal: 25 Cardiac: 96 FAST: 7 Lung: 46 Nerve: 5 Superficial: 4 Vascular: 14
Location (by patient)	ICU: 100 ED: 46 Ward: 10	ICU: 88 ED: 24 Ward: 8	ICU: 21 ED: 14 Ward: 5	ICU: 28 ED: 13 Ward: 8
Depth (STD, cm)	11.77 (3.48)	12.66 (3.47)	11.28 (4.64)	11.83 (4.02)

**Table 3:** K-fold cross-validation experiment data distribution averages and standard deviations across all folds (full results are provided in the Supplementary Materials and Table S4).

Class	Train			Validation			Test		
	Patients	Clips	Frames	Patients	Clips	Frames	Patients	Clips	Frames
**A-Lines**	202.1 (2.85)	575.4 (11.37)	147,880.8 (2814.89)	25.6 (2.07)	75.3 (9.29)	20,214 (2531.16)	25.3 (2.98)	72.3 (10.54)	18,677.22 (3345.67)
**B-Lines**	127.2 (3.48)	294.4 (12.48)	71,675.3 (3253.45)	12.3 (1.57)	24.2 (5.73)	5831.8 (1579.64)	15.5 (3.06)	35.4 (9.85)	8611.9 (2511.11)

**Table 4:** Summary metrics for a 10-fold cross-validation experiment on our local data and the external data inference.

Data Source	Metric	Accuracy	AUC	Precision	Recall/ Sensitivity	F1 Score	Specificity
**Local**	Mean	0.921 (SD 0.034)	0.964 (SD 0.964)	0.891 (SD 0.047)	0.858 (SD 0.05)	0.874 (SD 0.044)	0.947 (SD 0.036)
**External**	Value	0.843	0.926	0.886	0.812	0.847	0.878

## Data Availability

The details of the deep learning model used in this manuscript are available in the Supplementary Materials and the implementation can be found at this project’s GitHub repository: https://github.com/deepbreathe-ai/ab-line-classifer (last accessed on 25 October 2021). The patient data itself is not available for open-source sharing at this time but may be able to be made available in the future.

## Supplementary Materials

The following are available online at https://www.mdpi.com/article/10.3390/diagnostics11112049/s1, Figure S1: Effect of A vs. B line class and B line heterogeneity on B line prediction certainty, Figure S2: Effect of probe type, exam preset, and ultrasound vendor on A and B line prediction certainties for dataset 3 (external data, including homogenous and heterogenous clips) at the clip level, Figure S3: Effect of B line severity and heterogeneity on B line prediction false negative rate (FNR) using a clip level, average prediction of >0.5 for B lines, Figure S4: Influence of varying average clip prediction threshold on sensitivity and specificity for internal (A) and external data (B), Table S1: Graded qualification system for independent data labelling used to label local lung ultrasound data, Table S2: Visual summary of the model’s architecture, Table S3: Details of the runs comprising the Bayesian hyperparameter optimization, Table S4: K-fold cross validation experiment data distribution by patients, clips, and frames, Table S5: Full internal k fold validation results, Table S6: Clip-wise performance for B line detection across local and external data, Video S1: Video example of a homogeneous A line label on our lung ultrasound dataset. Horizontal reverberation artifacts from the pleural line (A lines) connote normal lung parenchyma and can be seen throughout the clip, uninterrupted, Video S2: Video example of a homogeneous fewer than 3 B lines label on our lung ultrasound dataset. A solitary, bright, ring-down artifact from the pleural line (B line) can be seen throughout the clip, Video S3: Video example of homogeneous moderate B line label on our lung ultrasound dataset. Multiple ring-down artifacts (B lines) can be seen throughout, occupying less than 50% of the total pleural line surface, Video S4: Video example of homogeneous severe B line label on our lung ultrasound dataset. Multiple ring-down artifacts (B lines) can be seen throughout, occupying more than 50% of the total pleural line surface, Video S5: Video example of a heterogeneous moderate B line label on our lung ultrasound dataset. Multiple ring-down artifacts (B lines) can be seen moving in and out of the field of view of the ultrasound image.
