# Supplementary material for: Automation of Lung Ultrasound Interpretation via Deep Learning for the Classification of Normal versus Abnormal Lung Parenchyma: A Multicenter Study
Source: Diagnostics (Basel). 2021 Nov 4;11(11):2049. doi: 10.3390/diagnostics11112049 (PMC8621216; doi:10.3390/diagnostics11112049)
Supplement: Supplementary file 1 [file diagnostics-11-02049-s001.zip › diagnostics-1442554 Supplementary Materials/diagnostics-1442554 Supplemenatry Materials.pdf]

Supplementary Materials

Supplementary Methods

Frame Based Classifier Training

Individual preprocessed frames were fed into the network as a tensor with dimensions 128×128×3. The input tensors were sequentially passed through the first 3 blocks (i.e. 10 layers) of VGG16. The 32×32×256 output of the final convolutional layer was passed to a 2D global average pooling layer. From there, it was subjected to dropout at a rate of 0.45, then passed to a 2-node fully connected layer with softmax activation.

Model training was split into 2 phases: feature extraction and fine-tuning. In feature extraction, all 10 VGG16 layers were frozen. The final layer was trained for 6 epochs using the Adam optimizer with a learning rate of 0.0003. During the fine-tuning phase, the weights comprising the third VGG16 block and output fully connected layer were allowed to train at a significantly lower learning rate for 9 epochs, using the RMSProp optimizer at a learning rate of 9.3×10^{-6}. The weights of the first two blocks of VGG16 were kept stagnant during this phase because they were previously trained on ImageNet to recognize low-level patterns in images, which also appear in lung ultrasound images. Once training was halted, the model weights yielding lowest loss on the validation set were restored, concluding a single training experiment. The best performing set of model weights on the validation set could then be used to evaluate model performance on the test set.

Hyperparameter Optimization Study

The results of the Bayesian hyperparameter optimization for the custom VGG16-based model architecture is displayed in Supplementary Table 3. The model was trained 6 times with varying results for a few hyperparameters. The metric of interest was AUC on a randomly selected test set. The number of runs was limited due to limited computational resources. The results informed the final selection of hyperparameters for future training experiments, including cross validation and the final training of the model.

Frame-Based Classifier Evaluation

To maximize our model performance, we conducted a hyperparameter search using Bayesian hyperparameter optimization.[26] The hyperparameters of interest were the learning rates for both the feature extraction and fine-tuning phase of training, the dropout rate, and the layer at which to unfreeze all subsequent layers during fine-tuning.

Model performance on our dataset was determined by conducting a 10-fold cross validation. A summary of the dataset partitions for our internal cross validation is shown in Table 4 (full table in the appendix - Supplementary Table 4).

A final training run was then performed with the selected architecture and hyperparameters to produce a model that would be used for clip-level classification and the validation on separate internal and external datasets.

Clip Averaging Prediction Algorithm

For clips where the ground truth is clearly and homogeneously represented across all frames, a clip averaging method would be an ideal way to determine the label of the clip. This method involves determining the average frame prediction across any given clip consisting of n frames, according to equation (1). The predicted class ŷ{0, 1}is then determined as the class with the greatest probability, as in Equation 2. By convention, ŷ=0 and ŷ=1 indicate A lines and B lines respectively.

Equation 1: $$p_{clip} = (1/n) \sum_{i=1}^{n} p_i$$

Equation 2: $$\hat{y} = argmax(p_{clip})$$

Given the common occurrence of heterogeneous clips in lung ultrasound (where B lines occur intermittently and form a minority of frames

Contiguous B line clip prediction algorithm

Equation 3 defines how a clip prediction is obtained, given the *n* B-line predicted probabilities from a clip. Recall that this method predicts B lines if there exists a group of consecutive frames for which the prediction probability provided by the model exceeds the classification threshold. This method can be applied in real time with negligible computational overhead by keeping a running count of contiguous frames for which the model's prediction probability exceeds the classification threshold *t*. If, at any time, the count climbs to the contiguity threshold *τ*, the prediction for the clip is set to B lines. If the count never reaches *τ*, the clip prediction is A lines. Algorithm 1 clearly defines this procedure. Note that this method could be generally applied using any function *f* that distinguishes frames containing A lines and B lines.

Training Hardware

All training experiments were conducted using an Intel® Core™ i9-10900K CPU at 3.70GHz and a NVIDIA® GeForce RTX™ 3090 GPU. The average and standard deviation inference runtime for a single frame on this hardware was 2.638ms and 0.485ms respectively (379 frames / second).

Algorithm 1

Require f: Function that receives LUS frame and outputs the probability that the frame contains B-lines (a trained neural network in the present work)
Require $t \in [0, 1]$: Classification threshold
Require $\tau \in \mathbb{N}$: Contiguity threshold

$c \leftarrow 0$ (Initialize maximum number of observed contiguous B line predictions)
$\hat{y} \leftarrow 0$ (Initialize clip prediction as "A lines")
while collecting frames **do**
    $x \leftarrow$ collect frame with probe
    $p_B \leftarrow f(x)$
    if $p_B \geq t$
        $c \leftarrow c + 1$
    else
        $c \leftarrow 0$
    if $c == \tau$
        $\hat{y} \leftarrow 1$ (Set clip prediction to "B lines")
        break
end while
return $\hat{y}$

Supplementary Results

Applying the clip-level averaging method to both the internal and external clip-based data sets, resulted in weak performance at the clip level for clips with heterogeneously present B lines and/or weakly positive B lines (Supplementary Figure 3). A more detailed portrayal of how clip averaging performed and the influence of data subtypes (vendors, presets, transducers) in this dimension of performance is found in supplementary figures 1&2, supplementary table 6.

The relationship of clip-average prediction levels to overall performance on our dataset can be seen in supplementary figure 4. It can be seen that clip averages of less than 0.5 generate the best overall performance for both the internal (0.15 clip-averaged prediction generating 85% sensitivity and specificity) and external (0.35 clip-averaged prediction generating 0.75 sensitivity and specificity). Averages outside these optimum points produce greater sensitivity (lower clip averaged threshold) or specificity (higher clip averaged thresholds).

Supplementary Figures

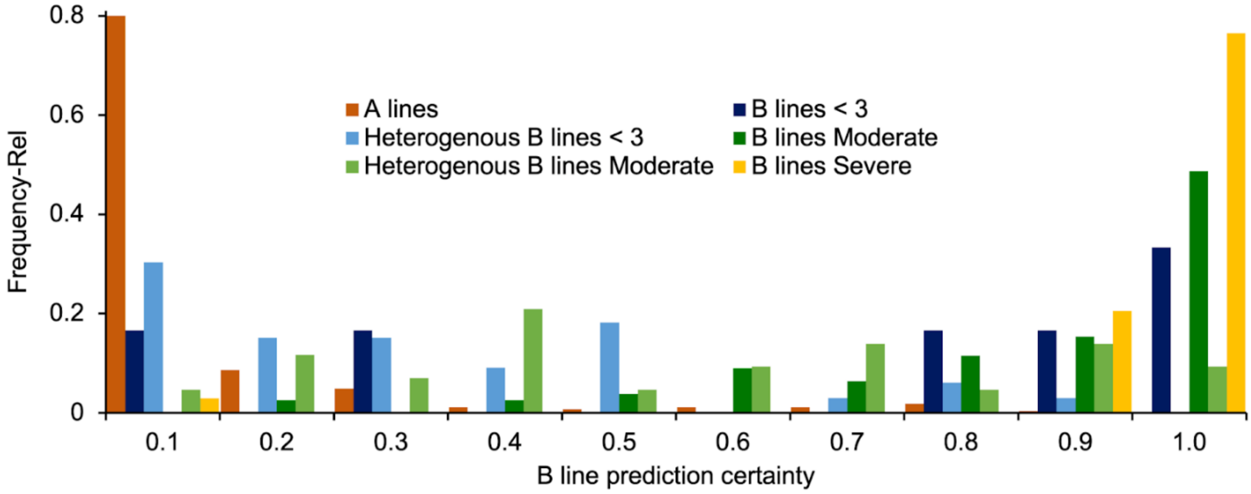

Supplementary Figure S1. Effect of A vs. B line class and B line heterogeneity on B line prediction certainty. Clips from dataset 2 (local data consisting of homogenous and heterogenous clips) were used. B line prediction certainties for each class were binned and the contents of each bin were normalized to the total number of clips in each class to determine the relative frequency (Frequency-Rel) and plotted. At the clip-wise level, both mild B lines and heterogeneity contribute to false negative B line predictions.

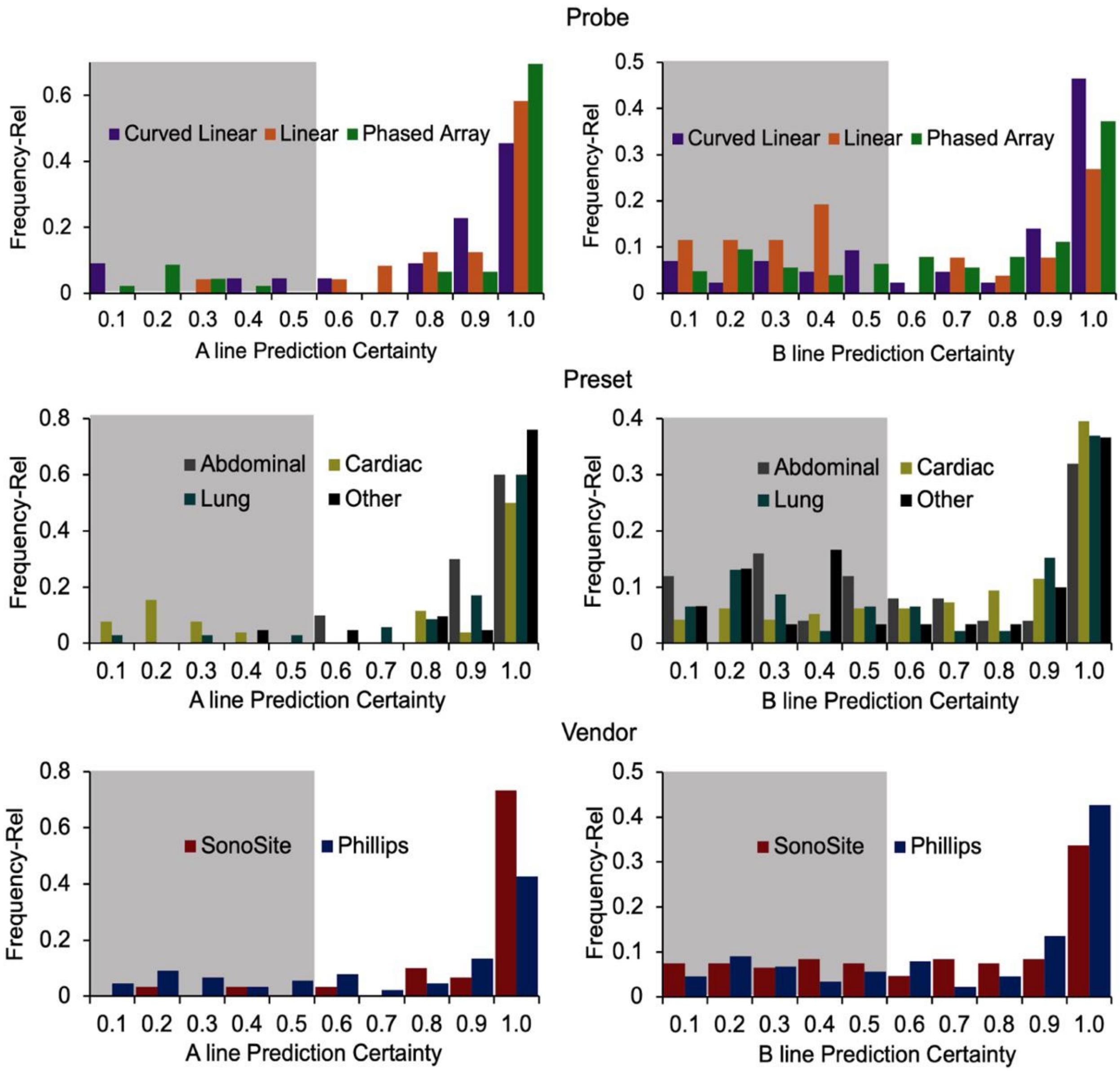

Supplementary Figure S2. Effect of probe type, exam preset, and ultrasound vendor on A and B line prediction certainties for dataset 3 (external data, including homogenous and heterogenous clips) at the clip level. A line prediction certainties for ground truth A line clips (left) and B line prediction certainties for ground truth B line clips (right) were arranged by probe type, exam preset and binned. The contents of each bin were normalized to the total number of clips in each class to determine the relative frequency (Frequency-Rel) and plotted. A prediction certainty of > 0.5 was the threshold for determining A or B lines. For ground truth A lines the curved linear probe, cardiac preset, and Philips machines resulted in lower A line prediction certainties and subsequently more incorrect predictions. For ground truth B lines the linear probe resulted in lower B line prediction certainties and subsequently more incorrect predictions.

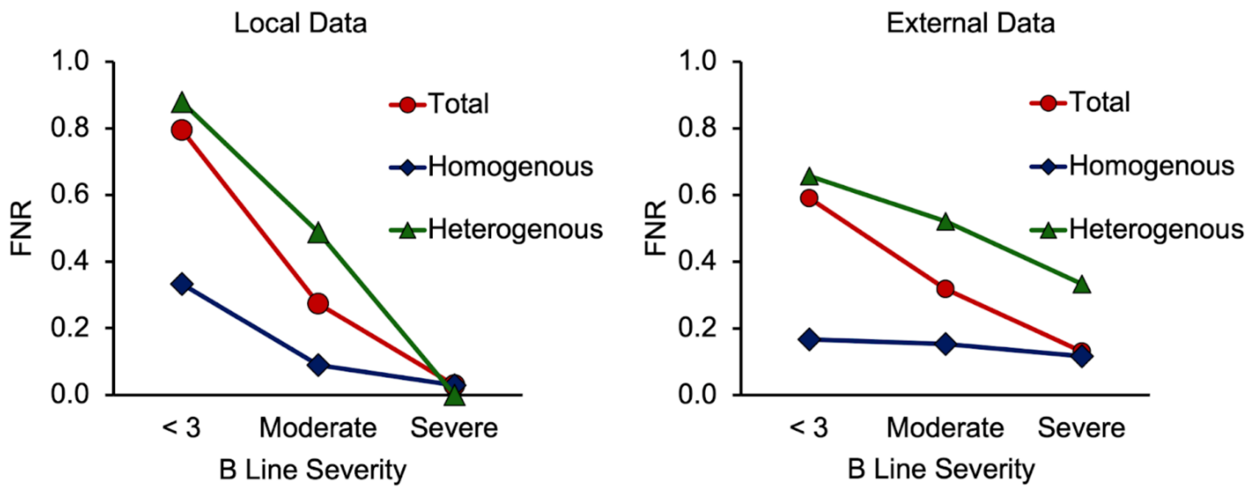

Supplementary Figure S3. Effect of B line severity and heterogeneity on B line prediction false negative rate (FNR) using a clip level, average prediction of > 0.5 for B lines. Data was then stratified according to whether the B lines were homogenous or heterogenous. In the absence of any thresholding outside of the 0.5 prediction norm for B lines, the classifier struggles with both lower B line burden and B line heterogeneity. This observation is consistent among local and external data.

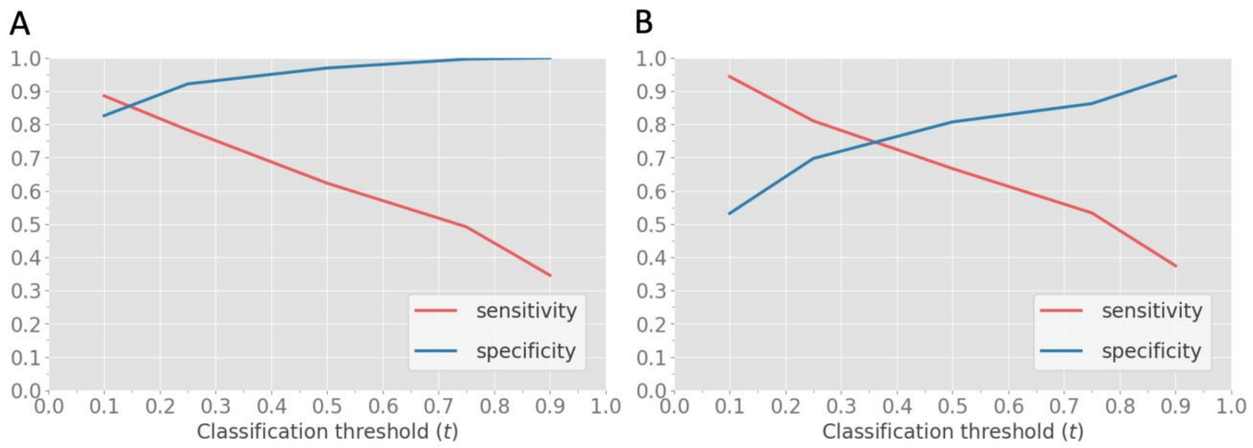

Supplementary Figure S4. Influence of varying average clip prediction threshold on sensitivity and specificity for internal (A) and external data (B).

Supplementary Tables

Supplementary Table S1. Graded qualification system for independent data labelling used to label local lung ultrasound data.

Level	Requirements	Ability
1	• Minimum of 3 hours of didactic and hands on lung ultrasound training workshop covering the acquisition and interpretation of lung ultrasound<br>• Thorough introduction to labelling software, completion of orientation guide and in-person walk through of labelling examples	• May label parenchymal views with oversight/overreading of all clips
2	• Requirements of level 1<br>• Completion of 500 labels of parenchymal views with > 90% accuracy	• Label parenchymal views independently with ability to request expert oversight ("request 2nd opinion" radio button)<br>• Label pleural views with oversight of all clips<br>• Review clips from level 1 team members (with backup support from expert team members)
3	• Requirements of level 2<br>• Completion of 750 pleural view labels with > 90% accuracy	• Label parenchymal and pleural views with ability to request expert oversight ("request 2nd opinion" radio button)<br>• Review clips from level 2 team members (with backup support from expert team members)
4	• Requirements of level 3<br>• Completion of 2500 parenchymal and 2500 pleural labels (> 98% accuracy of audited studies)	• Supervise and review efforts of levels 1-3<br>• Report directly to the project's expert supervisor

Supplementary Table S2. Visual summary of the model's architecture. The intermediate tensor sizes following the application of each layer in the model are detailed in the table below. In total, the model has 1,736,002 parameters.

Layer Number	Type	Block	Output Tensor Shape	Number of Parameters
0	Input	Input image	B × 128 × 128 × 3	0
1	2D Convolutional	VGG16 Block 1	B × 128 × 128 × 64	1,792
2	2D Convolutional	VGG16 Block 1	B × 128 × 128 × 64	36,928
3	2D Max Pooling	VGG16 Block 1	B × 64 × 64 × 64	0
4	2D Convolutional	VGG16 Block 2	B × 64 × 64 × 128	73,856
5	2D Convolutional	VGG16 Block 2	B × 64 × 64 × 128	147,584
6	2D Max Pooling	VGG16 Block 2	B × 32 × 32 × 128	0
7	2D Convolutional	VGG16 Block 3	B × 32 × 32 × 256	295,168
8	2D Convolutional	VGG16 Block 3	B × 32 × 32 × 256	590,080
9	2D Convolutional	VGG16 Block 3	B × 32 × 32 × 256	590,080
10	2D Global Average Pooling	Output Block	B × 256	0
11	Dropout	Output Block	B × 256	0
12	Fully Connected	Output Block	B × 2	514

Supplementary Table S3. Details of the runs comprising the Bayesian hyperparameter optimization. Each row gives the hyperparameter values sampled for a particular training run, along with the AUC on a test set randomly selected for each run. Note that the total number of epochs was held constant at 15; consequently, the number of epochs dedicated to fine-tuning was the difference between 15 and the number of epochs sampled for feature extraction.

Trial #	Learning rate for feature extraction	Learning rate for fine tuning	Dropout rate	Number of epochs for feature extraction	Test AUC
1	3.0×10^{-4}	2.0×10^{-6}	0.47	4	0.98
2	1.3×10^{-4}	8.4×10^{-6}	0.18	6	0.96
3	6.1×10^{-5}	3.9×10^{-6}	0.10	8	0.97
4	3.5×10^{-4}	9.3×10^{-6}	0.30	9	0.99
5	7.2×10^{-4}	2.0×10^{-6}	0.17	5	0.95
6	4.2×10^{-4}	1.2×10^{-6}	0.16	3	0.97

Supplementary Table S4. K-fold cross validation experiment data distribution by patients, clips, and frames.

Fold	Class	Train			Validation			Test		
		Patients	Clips	Frames	Patients	Clips	Frames	Patients	Clips	Frames
1	A-Lines	203	579	149882	28	89	23880	22	55	13010
	B-Lines	128	285	70581	9	25	5820	18	44	9718
2	A-Lines	207	579	149380	24	72	21900	22	72	15492
	B-Lines	120	270	65400	14	35	8938	21	49	11781
3	A-Lines	203	586	151562	22	62	17520	28	75	17690
	B-Lines	127	300	73159	14	20	4380	14	34	8580
4	A-Lines	198	572	146872	26	78	18600	29	73	21300
	B-Lines	132	305	74059	12	20	5580	11	29	6480
5	A-Lines	201	592	150052	28	74	19620	24	57	17100
	B-Lines	125	278	66499	13	24	5820	17	52	13800
6	A-Lines	200	557	143272	26	82	20340	27	84	23160
	B-Lines	131	305	74179	11	14	3420	13	35	8520
7	A-Lines	200	560	143752	24	75	19920	29	88	23100
	B-Lines	130	306	74959	13	23	4560	12	25	6600
8	A-Lines	200	581	148972	26	73	18840	27	69	18960
	B-Lines	127	296	72739	13	24	5700	15	34	7680
9	A-Lines	203	566	145792	28	87	24540	22	70	16440
	B-Lines	127	301	72319	11	29	7380	17	24	6420

**10**	**A-Lines**	206	582	149272	24	61	16980	23	80	20520
	**B-Lines**	125	298	72859	13	28	6720	17	28	6540

Supplementary Table S5. Full internal k fold validation results

Fold	Accuracy	AUC	F1 Score (A_lines)	F1 Score (B_lines)	Precision (A_lines)	Precision (B_lines)	Recall (A_lines)	Recall (B_lines)
1	0.8741	0.94	0.8907	0.8515	0.8852	0.8587	0.8962	0.8444
2	0.856	0.9312	0.873	0.8339	0.8752	0.8311	0.8707	0.8368
3	0.9178	0.9623	0.9388	0.8748	0.9411	0.8704	0.9365	0.8791
4	0.9543	0.989	0.9703	0.9009	0.9669	0.9115	0.9737	0.8906
5	0.9268	0.9748	0.9356	0.9154	0.9124	0.9469	0.9599	0.8859
6	0.9628	0.9939	0.9749	0.9287	0.9641	0.959	0.9858	0.9004
7	0.9544	0.984	0.9707	0.8973	0.9704	0.898	0.9709	0.8965
8	0.9218	0.9785	0.9451	0.8647	0.9458	0.8631	0.9444	0.8663
9	0.947	0.9864	0.9639	0.9008	0.9459	0.9502	0.9825	0.8562
10	0.8959	0.9649	0.9327	0.7701	0.9146	0.8261	0.9516	0.7213

Supplementary Table S6. Clip-wise performance for B line detection across local and external data. At the clip-wise level with a prediction certainty defined by > 0.5, the false positive rate (FPR) was higher for the external data, while the false negative rate (FNR) for the external data was similar to local data.

	Local data	External data
FPR ($\frac{FP}{FP+TN}$)	0.03	0.14
FNR ($\frac{FN}{FN+TP}$)	0.38	0.34
